# Evaluation of Ultra-High Pressure Homogenization Treatments to Ensure the Microbiological Safety and Immunoglobulin Preservation in Donor Human Milk

**DOI:** 10.3390/foods14081310

**Published:** 2025-04-09

**Authors:** Kimia Jalali, Belén Pastor-Villaescusa, Katherine Flores-Rojas, Vanessa Pleguezuelos, Francisco J. Pérez-Cano, Àngels Franch-Masferrer, Antonio J. Trujillo-Mesa, M. Manuela Hernández-Herrero, Artur X. Roig-Sagués

**Affiliations:** 1 Centre d’Innovació, Recerca i Transferència en Tecnologia dels Aliments, TECNIO CERTA-UAB, Departament de Ciència Animal i dels Aliments, Universitat Autònoma de Barcelona, 08193 Bellaterra, Spain; kimia.jalali@uab.cat (K.J.); toni.trujillo@uab.cat (A.J.T.-M.); manuela.hernandez@uab.cat (M.M.H.-H.); 2 Metabolism and Investigation Unit, Maimonides Institute of Biomedicine Research of Córdoba (IMIBIC), Reina Sofia University Hospital, University of Córdoba, 14001 Córdoba, Spain; katherine1.flores@gmail.com; 3 Primary Care Interventions to Prevent Maternal and Child Chronic Diseases of Perinatal and Developmental Origin (RICORS), RD21/0012/0008, Instituto de Salud Carlos III, 28040 Madrid, Spain; 4 Spanish Network in Maternal, Neonatal, Child and Developmental Health Research (RICORS-SAMID, RD24/0013/0007) Instituto de Salud Carlos III, 28040 Madrid, Spain; 5 Catalan Department of Health, Banc de Sang i Teixits (BST), 08005 Barcelona, Spain; vpleguezuelos@bst.cat; 6 Physiology Section, Department of Biochemistry and Physiology, Faculty of Pharmacy and Food Science, Universitat de Barcelona (UB), 08028 Barcelona, Spain; franciscoperez@ub.edu (F.J.P.-C.); angelsfranch@ub.edu (À.F.-M.); 7 Instituto de Investigación en Nutrición y Seguridad Alimentaria (INSA-UB), 08921 Santa Coloma de Gramenet, Spain

**Keywords:** human milk, holder pasteurization, UHPH, pathogen inactivation, immunoglobulins, bioactive components

## Abstract

Most donor human milk (HM) banks use Holder pasteurization (HoP) to ensure microbiological safety, although it can degrade essential bioactive factors for newborns. This study evaluates the innovative ultra-high-pressure homogenization (UHPH) technology as a potential alternative. *Listeria innocua*, *Staphylococcus carnosus*, *Franconibacter helveticus* (formerly named *Cronobacter helveticus*) and *Escherichia coli* strains were used as surrogates for common HM pathogens according to European Milk Bank Association (EMBA) guidelines, to evaluate the efficacy of new technologies. A reconstituted powder milk formula inoculated with these strains was used to determine the most efficient conditions (those to achieve a lethality of ≥5 Log), applying treatments from 150 to 300 MPa. These treatments were later validated using inoculated HM with the same strains. Immunoglobulin (sIgA, IgG, IgM) retention was also evaluated and compared with HoP. Results showed that UHPH treatments at 200 MPa achieved a lethality > 5 Log for all strains, except for *St. carnosus*, which required 250 MPa for complete inactivation in HM. Unlike HoP, UHPH at 200 and 250 MPa did not significantly reduce the basal concentration of sIgA, IgG, or IgM compared with raw HM. These findings suggest UHPH as a promising alternative to HoP, maintaining both microbiological safety and immunological quality.

## 1. Introduction

Human Milk Banks (HMBs) play a fundamental role in providing optimal nutrition for premature and sick newborns who cannot or are partially breastfed. The administration of donor human milk (HM) has become an increasingly common practice. According to the World Health Organization (WHO), it is considered the first alternative when, due to various circumstances, the mother’s own milk is not fully or partially available. This applies to both premature infants and those facing health challenges [1]. HMBs play a fundamental role as they allow the collection, processing, analysis, and proper distribution of HM. So far, recommendations from the European Milk Banking Association (EMBA) and concretely those established by Calvo et al. [2] have been followed in Spain for all the HMB operations. Fortunately, donor HM has recently been incorporated into EU regulation 2024/1938 under the “Regulation on standards of quality and safety for substances of human origin intended for human application” [3]. As a result, its collection, processing, analysis, storage, and distribution are now governed by this EU regulation. In most of the European HMBs, milk is kept frozen after expressing and subsequently subjected to a low-temperature, long-term thermal treatment (62.5 °C, 30 min), called “Holder pasteurization” (HoP). This method is considered the standard treatment to guarantee the microbiological safety and nutritional value of HM, as it inactivates bacteria in vegetative form, fungi, and some viruses [4]. Nevertheless, this treatment can compromise the integrity of various bioactive compounds, such as polyunsaturated fatty acids, phospholipids, cytokines, immunoglobulins, hormones, and growth factors [5,6] that play a crucial role in the visual, motor, and cognitive development of infants, as well as in preventing infections in premature newborns [7]. Due to the limitations of HoP in processing the HM, the EMBA and the Nutrition Committee of the European Society of Pediatric Gastroenterology, Hepatology and Nutrition (ESPGHAN) recently recommended that future research should be directed toward the development and evaluation of different pasteurization techniques to optimize the microbiological safety and maintain the biological and nutritional quality of HM [8].

Ultra-high-pressure homogenization (UHPH) is an emerging technology with multiple applications in fluid processing, which has been proposed as an alternative to thermal pasteurization for liquid foods, such as milk and dairy products, fruit juices, and vegetable milks, among others [9,10,11,12]. The UHPH is based on the principles of conventional homogenization used in the dairy industry but using pressures of up to 350 MPa. UHPH extends the shelf life of foods by inactivating microorganisms due to the shear forces, impact, pressure, cavitation, and other factors that occur at the homogenization valve. Depending on the applied conditions, it can result in microbiologically stable products suitable for chilled or room temperatures [12]. UHPH also enhances food matrices’ functionality by increasing emulsion capacity and stability, without affecting nutritional value and sensory characteristics [13]. Although UHPH is not typically considered as a heat treatment, temperature increases occur during processing as the product passes through the valve due to the applied forces previously described. The temperature increases with increasing pressure, but with ultra-short residence times (<0.7 s) [14]. This brief exposure to high temperatures minimizes thermal effects on bioactive components while still contributing to microbial inactivation alongside the other physical forces developed at the valve [12].

There is little literature evaluating the positive effect of UHPH treatment at 200 MPa at an inlet temperature of 20 °C on HM in inactivating non-spore-forming microorganisms and its impact on bioactive components [15]. Moreover, there is no information about the effectiveness of UHPH in inactivating pathogenic microorganisms usually present in HM, and there are scarce data on the efficacy of this treatment on bioactive compounds.

The main objective of this study is to propose alternative treatments to HoP based on the use of UHPH to ensure microbiological safety while preserving the bioactive quality of the donor HM, specifically its immunological components.

## 2. Materials and Methods

### 2.1. Preparation of Bacterial Strains

According to the EMBA recommendations [8], *Listeria monocytogenes*, *Staphylococcus aureus*, and *Cronobacter sakazakii* should be considered in the microbiological validation of a new pasteurization process. Since the UHPH treatments were performed in a pilot plant that does not meet biosafety level 2 requirements, we selected non-pathogenic, biosafety level 1 surrogates of these microorganisms. Accordingly, *Listeria innocua* (CECT910), *Staphylococcus carnosus* (CECT4491), and *Franconibacter helveticus* (formerly named *Cronobacter helveticus*) (CECT8568) were used as surrogates. These strains were previously used to evaluate the efficacy of UHPH in cow milk [16,17,18], except the strain of *C. helveticus*. An *Escherichia coli* strain (CECT423) was also included to cover other potentially pathogenic bacteria of the Enterobacteriaceae family. All the strains were obtained from the Spanish Type Culture Collection (CECT, Universitat de València, Spain) as a freeze-dried culture. They were first recovered in Tryptone Soy Broth (Oxoid, Basingstoke, UK), incubated overnight at 37 °C, and later streaked on a Petri plate with Tryptone Soy Agar (Oxoid) and incubated for 24 h at 37 °C. After testing the purity of cultures, colonies were transferred to 0.9% (*w*/*v*) saline solution to achieve a final concentration above 8 Log CFU/mL.

### 2.2. Preparation of Infant Formula Milk and Collection of Human Milk Samples

To optimize process parameters while minimizing the use of donor HM, a reconstituted infant formula (IF; type 1: from 0 to 6 months of age) was used as a model system. This allowed us to assess the efficacy of the technological treatments on inoculated microorganisms before applying them to donor HM. IF was reconstituted according to the manufacturer recommendations before inoculation. Lethality results were later validated using donor HM to evaluate the impact of the selected treatments on the microbial strains as well as on the immunological components of HM.

HM samples were obtained through the HMB of the Reina Sofía University Hospital (RSUH) of Córdoba and from the *Banc de Sang i Teixits* (Blood and Tissue Bank, BST) of Catalonia. These HMBs comply with ethical donor data processing and sample manipulation standards. The inclusion criteria for the donor women aligned with the standard guidelines used by these HMBs, to select the most appropriate donors, considering their health, lifestyle, and potential factors that might influence the milk quality. However, for the analyses of microbiological validation, discarded HM from women whose milk did not meet the safety and quality standards required for distribution to newborns, as it was stored at −20 °C for >1 month after being expressed, was used. Furthermore, for the analyses of immunological components, HM samples were collected from six donors who had given birth to both preterm and term infants. The samples were obtained between the first and third months of lactation and were stored frozen at −20 °C for no more than six months. These criteria were chosen to ensure sufficiently high immunoglobulin (Ig) levels in raw HM for accurate quantification [19]. All samples were obtained, at a minimum, after 15 days postpartum (mature milk).

The HM from different donors was stored at −80 °C in the HMBs and transported to the laboratory in closed containers with dry ice. The samples remained at −20 °C until preparing in pools before treatments.

In the case of microbiological analysis, different HM samples from the same donor were pooled. For immunological analysis, the pool was performed using the HM samples from the six donors. The pool was distributed for three trials in each treatment.

### 2.3. Ethical Aspects

This study was performed in accordance with the Declaration of Helsinki and was approved by the Institutional Hospital Ethical Committee (RSUH, Córdoba, Spain, 25 July 2023; human ethics approval number 5344_2020). Donor HM samples from the HMB of Catalonia were obtained through the BST Biobank following approval of the Agreement for Biological Samples and/or Clinical Data for Biomedical Research (CEIm Hospital U. Vall d’Hebron. PR(CS) 499/2023). HM donors were informed, and signed consent was obtained before data and sample collection.

### 2.4. Treatments

#### 2.4.1. Holder Pasteurization (HoP)

HM samples were distributed in 220 mL bottles and submitted to 62.5 °C for 30 min followed by a rapid cooling to 4 °C using a HoP equipment (Sterifeed ECO S90, Devon, UK). After HoP treatment, samples were kept at ≤4 °C for less than 24 h until microbiological analysis or at 4 °C until further processing for immunological component quantification.

#### 2.4.2. Ultra-High-Pressure Homogenization (UHPH)

The UHPH treatments were performed with a 60 L/h Ypsicon high-pressure homogenizer (model A60 Ypsicon Advanced Technologies, S.L., Barcelona, Spain). This includes a high-pressure valve capable of withstanding 350 MPa. Two spiral-type heat exchangers (Garvía, Barcelona, Spain) minimize the temperature retention after treatment. The inlet temperature (Ti), the temperature before reaching the homogenizing valve (Tv), as well as the final temperature of the sample after passing through the heat exchanger (Tf), were monitored throughout the experiments. UHPH treatments ranged from 150 to 300 MPa at a Ti of 13–20 ± 2 °C. Once treated, samples were collected aseptically in a Telstar PCR Mini-V biosafety cabinet (Telstar) in sterile containers and kept refrigerated (4 °C) until analysis.

### 2.5. Microbiological Analysis of the Milk Samples and Lethality Determination

Before treatments, both IF and HM were inoculated under aseptic conditions using a laminar flow cabin, with a cocktail of the selected strains to achieve a final load above 5 Log CFU/mL. For HM, samples were defrosted, mixed, and inoculated. The mixture in both models was kept at 4 °C before the treatments.

Ten-fold dilutions were made from each sample in buffered peptone water (Oxoid). Aliquots of each dilution were plated in Petri plates containing selective culture media: Brilliance Listeria agar (Oxoid) incubated at 30 °C for 48 h for *L. innocua*; Mannitol Salt Agar (Oxoid) incubated at 30 °C for 48 h for *St. carnosus*; Chromogenic Coliform Agar (CCA) (Oxoid) incubated at 37 °C for 24 h for *E. coli*; Brilliance sakazakii Agar (Oxoid) incubated at 37 °C for 24 h for *Cr. helveticus*. The lethality caused by the UHPH treatments on the inoculated microorganism was estimated with Equation (1):

(1) $$\mathrm{Lethality}=-{Log}_{10}\left(\frac{N}{N_{0}}\right)$$

where *N_0_* is the initial number of vegetative cells present in the samples before the treatments and *N* is the number of remaining viable cells after the treatments, both expressed as CFU/mL.

### 2.6. Determination of the Inactivation Kinetics and Effect of Treatment Variables

Different statistical models from the results obtained with HM after UHPH treatments were elaborated based on the lethality as a response variable and different explanatory or independent variables: (1) *Log C*, corresponding to the quantity of inoculated microorganisms expressed as Log CFU/mL; (2) pressure applied (*P*) and valve temperature (*Tv*). Three mathematical models were evaluated: linear (Equation (2)), quadratic (Equation (3)), and exponential (Equation (4)).

(2) Lethality=β0+β1·Log C+β2·P+β3·Tv

(3) $$Lethality=\beta 0+\beta 1\cdot Log\;C+\beta 2\cdot P+\beta 3\cdot Tv+\beta 4\cdot P^{2}+\beta 5\;\cdot {Tv}^{2}+\beta 6\;P\;x\cdot Tv$$

(4) $$Lethality=e^{\beta 0+\beta 1\;\cdot \;Log\;C+\beta 2\;\cdot \;P+\beta 3\cdot \;Tv}$$

### 2.7. Immunological Components Quantification in HM Samples

#### 2.7.1. Preparation of HM Samples for Immunoassays

All HM samples maintained at 4 °C were processed within 2 h after treatment. To avoid interference of milk fat content with the immunoassays, lactic serum separation was performed as previously described [19]. Briefly, the milk fatty layer and cellular elements were removed by centrifugation at 800× *g* for 10 min at 4 °C and the intermediate aqueous phase was aliquoted to avoid repeated freeze–thaw cycles and stored at −80 °C until Ig analysis.

#### 2.7.2. sIgA, IgG, and IgM Immunoassays

The quantification of secretory IgA (sIgA) was performed in the lactic serum by a Human Sandwich IgA ELISA Kit (Invitrogen, Thermo Fisher Scientific, Vienna, Austria) following the manufacturer instructions and validated for quantification in mucosal fluids. Data were analyzed by Multiskan Ascent v2.6 software (Thermo Fisher Scientific), with an assay sensitivity of 1.6 ng/mL.

The quantification of IgG and IgM was also conducted in the aqueous phase (lactic serum) by ProcartaPlex^TM^ Multiplex Immunoassay (Thermo Fisher Scientific) as in previous studies on breast milk [20]. Briefly, magnetic microsphere beads labelled with antibodies specific for a single target protein were used. The addition of the beads of interest in the plate led to the analysis of multiple targets in a single well. Finally, the plate was run on a Luminex Instrument and analyzed in a ProcartaPlex Analyst Software 1.0 (MAGPIX^®^ analyzer, Luminex Corporation, Austin, TX, USA) at the Cytometry Unit of the Scientific and Technological Centers of the University of Barcelona (CCiTUB). Assay sensitivity was <1 ng/mL for IgG and 6.41 ng/mL for IgM.

The Ig isotype concentrations were set up as 100% for the untreated HM samples (raw HM) and expressed as a percentage relative to their corresponding basal values after each treatment.

### 2.8. Statistical Analysis

Three independent trials were performed for each experiment, and two different samples were taken (n = 6). An analysis of variance (ANOVA) and Tukey test were used to compare results between treatments and/or matrices in the microbiological assays. This analysis was performed using the R system for statistical computation (R Foundation for Statistical Computing, version R-4.4.2 for Windows, Vienna, Austria 2014, http://www.R-project.org, accessed on 10 February 2025). Data were expressed as mean ± standard deviation. Regression analyses were also performed with the R system for statistical computation using different functions for linear, polynomial, or non-linear models. Significance tests for independent variables were assessed using *p* < 0.05.

Statistical analysis of the Ig isotype concentrations was carried out by the software IBM Statistical Package for the Social Sciences (SPSS, version 22.0, Chicago, IL, USA). To evaluate the homogeneity of variance and the distribution of the data, Levene’s and Shapiro–Wilk tests were carried out, respectively. As Ig concentrations were not normally distributed, a non-parametric test was used. A Kruskal–Wallis unpaired test was performed, and the significant effect of the treatment was then analyzed by the Mann–Whitney U test. Differences were considered to be significant at *p* < 0.05.

## 3. Results and Discussion

### 3.1. Effect of UHPH on Pathogen Strains Inoculated in Reconstituted Powdered Infant Formula and Human Milk

Table 1 shows the results for the parameters analyzed during the application of UHPH treatments. The maximum temperature recorded in the valve (Tv) increased with the treatment pressure, reaching a peak of 121 °C at 300 MPa. While this temperature exceeds that used in HoP (62.5 °C), the sample is exposed to this high temperature for an extremely short period (estimated at <0.7 s), compared to HoP, where the sample is kept for a minimum of 30 min.

**Figure 1:**
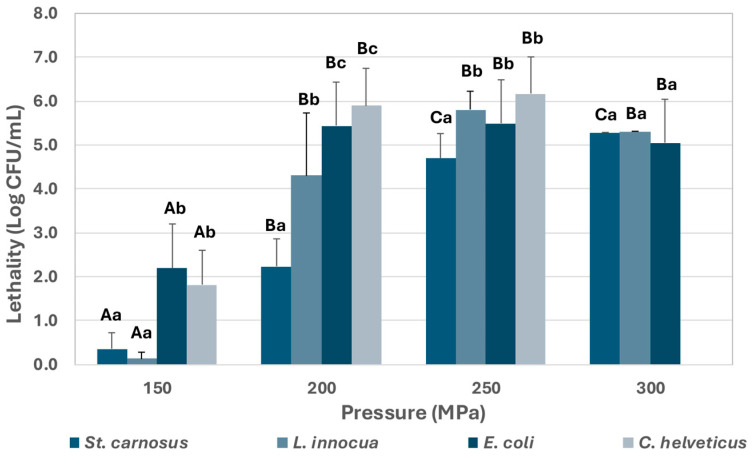
Lethality caused by UHPH treatments at pressures of 150, 200, 250, and 300 MPa in reconstituted powdered IF. The results are expressed as the mean of the Log CFU/mL ± standard deviation. Different capital letters in the data columns indicate significant differences (*p* < 0.05) between treatments at different pressures for the same microorganism. Different lowercase letters indicate significant differences (*p* < 0.05) between microorganisms for the same treatment.

**Figure 2:**
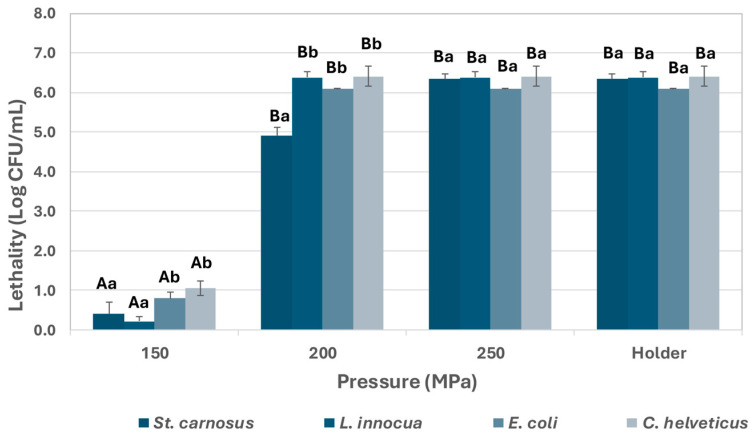
Lethality caused by UHPH treatments at pressures of 150, 200, and 250 MPa and by HoP (62.5 °C 30 min) in HM. The results are expressed as the mean of the Log CFU/mL ± standard deviation. Different capital letters in the data columns indicate significant differences (*p* < 0.05) between UHPH treatments at different pressures and HoP for the same microorganism. Different lowercase letters indicate significant differences (*p* < 0.05) between microorganisms for the same treatment.

Figure 1 and Figure 2 present the effect of UHPH treatments on the inoculated microorganisms in both the reconstituted powdered IF and the HM, respectively. Figure 2 also shows the lethality observed after applying the HoP treatment according to the usual HMB procedure. As shown, UHPH treatments at 150 MPa demonstrated a very low efficacy in both IF and HM, substantially below the effectiveness of the HoP treatment, which achieved the inactivation of all viable cells of the inoculated microorganisms in HM, with a lethality greater than 5 Log CFU/mL. The lethality caused by UHPH treatments increased significantly when the pressure was increased to 200 MPa, although *S. carnosus* showed significantly greater resistance than the rest of the microorganisms. This resistance, however, was lower when the microorganism was inoculated into the HM. *L. innocua* also showed more resistance to UHPH treatments at 200 MPa than *C. helveticus* and *E. coli* in IF but did not differ when inoculated in HM. No significant differences were observed between the results of the treatments at 250 MPa and 300 MPa in IF, obtaining a complete inactivation of all inoculated strains. Consequently, the 300 MPa treatment was not applied to HM.

Temperature is an important parameter to control during UHPH processing, as in addition to the possible lethal effect on microorganisms, it also affects enzymatic inactivation [21]. The maximum temperature reached usually depends on the initial temperature of the fluid, the composition of the matrix, and the pressure applied. There is a linear relationship between the applied pressure and the temperature of the fluid after passing through the homogenization valve. This is due to the increased fluid energy from exposure to the forces of turbulence, impact, shear, and cavitation, resulting in the temperature rising by 16 to 20 °C per 100 MPa [14,22]. However, the temperature increase in this study was greater, as the valve increased between 25 and 30 °C per 100 MPa from the Ti (20 or 13 °C, respectively) (Table 1). To minimize the effect of temperature, UHPH equipment usually includes a heat exchanger at the valve outlet to ensure immediate cooling of the milk. This reduces the time in which milk is kept at high temperatures, which has been estimated at less than 0.7 s [14]. Because of this, the heat stress caused by these treatments is much lower than that caused by conventional pasteurization or sterilization heat treatments. Considering the average D-values and z-values determined from the data collection analyzed by van Asselt and Zwietering [23], the maximum reductions that could be expected at the maximum temperatures reached in UHPH treatments are greater than those that could be simply attributed to heat treatment. As shown in Table 2, at a temperature of 75 °C, the maximum temperature reached in the valve in treatments at 200 MPa in HM, it would take 0.3 s to achieve a reduction of 1 Log of *C. sakazakii*, 1.01 s in the case of *L. monocytogenes*, 4.17 s for *S. aureus*, and 4.33 s in the case of *E. coli*. However, the mean reductions obtained by UHPH treatment were much higher. In fact, in the case of *S. carnosus*, the most resistant microorganism, the reduction obtained was almost 5 Log in HM.

Only one study has been published to date evaluating the use of UHPH for HM decontamination [15]. This study evaluated the effect of UHPH treatments of 200, 250, and 300 MPa, introducing the HM at 20 °C, on the counts of the total aerobic microbiota and Enterobacteriaceae naturally present in HM. The reductions reported at 200 MPa were about 3 Log of the total counts, and *Enterobacteriaceae*, which started from an initial level of about 4 Log, were no longer detected. However, no specific data have been published on the effect of UHPH on pathogenic microorganisms or surrogates in HM. Studies in which UHPH has been applied to the milk of other species, especially cow milk, are available. Briñez et al. [16] reported reductions slightly above 3.5 Log in two strains of *E. coli* inoculated in whole cow’s milk treated by UHPH at 300 MPa when milk was introduced at 6 °C. These results are considerably lower than those observed in our study, where the lethality of *E. coli* exceeded 5 Log at 250 MPa, with a Ti of 13 °C. In another piece of research [17], after applying the UHPH treatment to milk inoculated with the same *L. innocua* strain used in this study, lethality barely reached 3.5 Log, which was much lower than that observed at low pressures in the present study. Similar results were described for *S. aureus* and *S. carnosus* [18]. In the specific case of *S. carnosus*, no significant reduction was observed after 300 MPa treatment, indicating the high resistance of this strain. However, in this study, the *S. carnosus* strain showed itself to be much more resistant than the other evaluated strains; an average reduction above 2 Log was achieved in the treatments at 200 MPa and greater than 4.5 Log at 250 MPa when inoculated in IF. The lethality increased by about 5 Log at 200 MPa and exceeded 6 Log at 250 MPa when the HM was treated. This higher resistance of *Staphylococcus* strains to UHPH has been confirmed more recently by Dong et al. [24], reported in cow’s milk: after UHPH treatment at 200 MPa with a Ti of 40 °C, *E. coli* counts were reduced by 3.67 Log while the *S. aureus* only decreased by 1.02, showing that milk could provide a protective effect for *S. aureus* against UHPH. In fact, they observed that the cellular structure of *E. coli* was destroyed after the treatments, while no obvious damage was found in those of *S. aureus*.

Differences in the reported results of similar treatments on the same microorganisms or even on the same strains might be due to the different performances of the UHPH equipment used. In the case of the studies conducted by Briñez et al. [16,17,18], Stansted Fluid Power Ltd. equipment (model FPG7400H, Harlow, UK) with a double valve made of ceramic material was used, while in this work, more modern equipment with a different valve design (Ypsicon model A60, Barcelona, Spain) was used. The design of the homogenization valve is the core of the UHPH equipment and different valve designs exert different levels of shear on the fluid [25].

### 3.2. Mathematical Modeling of Treatments

Table 3 presents the mathematical models based on the lethality of different strains inoculated in HM. The response and various explanatory variables were analyzed to identify the model that best predicted values closest to the observed data. To determine the optimal model, several statistical parameters were considered, prioritizing the following: (1) the adjusted R^2^, which should be as close to 1 as possible; (2) the root mean square error (RMSE), which should be minimized; (3) the model with the fewest explanatory variables to enhance simplicity and interpretability. Based on these criteria, the linear model was identified as the best fit for HM, although the quadratic model also demonstrated a very good fit. In contrast, the exponential model exhibited the poorest performance across all cases.

### 3.3. Effect of UHPH on Immunoglobulin Isotype Concentrations in Human Milk

Since the treatment at 150 MPa was ineffective for microbial inactivation and the 300 MPa treatment showed similar efficacy to 250 MPa, these two conditions were not included in the immunological assessment, as they were not considered optimal.

The concentrations of sIgA, IgG, and IgM from the analyzed HM, corresponding to the 1st–3rd month of lactation, were considered raw values, representative of those from donor HM (Table 4). To inactivate pathogenic microorganisms, donor HM is pasteurized before its conservation and distribution, although it is known that this process also affects some of the nutritional and biological properties [6], as a consequence of the heat treatment. The primary limitation to the widespread use of donor HM for feeding preterm infants is the loss of certain biologically active components, including immunological factors, due to HoP [26]. Actually, it is important to highlight that Ig content has a critical role in the protective capacity of HM against infection [27].

In this study, HoP significantly reduced the proportion of the main Ig isotypes in HM, decreasing all three assessed Ig types by approximately 50% (*p* < 0.05 vs. raw HM values). Losses of the main three Igs contents after HoP treatment have been widely reported [28]. Some studies have reported even greater reductions after applying HoP, with IgA levels decreasing by up to 60% [29], IgG by 79% [30], and IgM by as much as 100% [30].

In contrast, UHPH treatments both at 200 and 250 MPa avoided such impact and did not significantly reduce the concentrations of sIgA, IgG, or IgM compared with raw HM. Overall, both UHHP treatments were demonstrated to maintain higher proportions (in most cases retaining over 90% of the raw values for all Ig isotypes) than their pasteurized counterpart HM (*p* < 0.05 vs. HoP). As mentioned, only one study has applied UHPH technology to donor HM, evaluating its efficacy in both HM decontamination and bioactive property preservation [15]. The authors reported promising results for Ig retention, specifically at 250 MPa and a Ti of 20 °C, with IgA, IgG, and IgM retention rates of 71.5%, 104%, and 71%, respectively, compared to 52%, 66.5%, and 27% with HoP [15]. However, 300 MPa pressures resulted in Ig losses comparable to those observed with HoP. Based on our findings, applying lower pressures in UHPH technology may be sufficient to achieve microbiological safety while better preserving Ig concentrations present in raw HM. Additionally, maintaining a lower Ti (13 °C) could further minimize thermal damage to heat-sensitive HM components, enhancing their retention.

It is well known how the maternal-milk-delivered fraction of Igs provides passive immunity to the infant [31]. Concretely, sIgA, the predominant Ig in HM, serves as the first line of defense in the intestine by preventing pathogen binding and neutralizing toxins, bacteria, and viruses [32]. IgG activates the complement cascade to eliminate pathogens and provides mucosal protection against viral infections [33], while IgM enhances defense by opsonizing antigens for complement fixation and destruction [34]. Given the need to enhance the properties of pasteurized donor HM for optimal feeding of infants in neonatal intensive care units, there is a growing demand for alternative processing methods that better preserve its bioactive components.

## 4. Conclusions

This study highlights the advantages of UHPH as a promising technology that ensures microbiological safety while minimizing the loss of Igs in HM—essential factors in providing immune protection to preterm and vulnerable infants. UHPH treatment at 200 MPa achieved a reduction greater than 5 Log for most of the inoculated microorganisms, with the exception of *S. carnosus*, which showed a reduction close to this level. Complete inactivation of all tested microorganisms was achieved at 250 MPa. Importantly, both UHPH treatments preserved sIgA, IgG, and IgM levels in HM, in contrast to the significant losses observed following HoP. Nevertheless, further research is necessary to optimize processing parameters, evaluate the impact on other bioactive components, and confirm the long-term safety and feasibility of implementing UHPH in routine milk bank operations.

## Figures and Tables

**Table 1:** Valve temperature and pressure changes during UHPH processing of reconstituted powdered infant formula and human milk.

Matrix	UHPH (MPa)	Tv (°C)	Ti (°C)	Tf (°C)
Infant formula	151.67 ± 3.66	60.20 ± 2.21	21.47 ± 0.86	18.24 ± 1.34
	201.22 ± 2.21	76.89 ± 8.04	20.93 ± 1.54	18.13 ± 1.12
	251.41 ± 3.81	88.35 ± 8.37	20.94 ± 1.52	17.56 ± 1.17
	300.50 ± 2.12	121.00 ± 0.00	24.95 ± 0.07	17.60 ± 0.14
Human milk	153.29 ± 2.81	60.86 ± 1.86	13.16 ± 0.08	18.21 ± 0.20
	204.14 ± 2.48	75.43 ± 1.72	13.40 ± 0.00	18.41 ± 0.07
	251.44 ± 1.94	85.22 ± 0.44	13.32 ± 0.20	15.89 ± 1.27

Data are presented as the mean value ± standard deviation. Tf = final product temperature; Ti = inlet temperature; Tv = valve temperature; UHPH = ultra-high-pressure homogenization.

**Table 2:** Mean D-values at reference temperature (70 °C) and estimation of D-value at valve temperature for 200 MPa UHPH treatments (75 °C).

Microorganism	Z Value (°C)	D_70°C_ (s)	D_75°C_ (s)
*Listeria monocytogenes*	7.0	5.23	1.01
*Staphylococcus aureus*	8.8	15.42	4.17
*Cronobacter sakazakii*	6.3	1.85	0.30
*Escherichia coli*	10.6	12.83	4.33

D_75°C_ was estimated according to van Asselt and Zwietering [23] data with the equation LogD = LogDref − (T − Tref)/Z.

**Table 3:** Mathematical models depending on the lethality UHPH treatments of different bacteria strains obtained in human milk.

Microorganism	Models	R^2^ adj	RMSE
*St. carnosus*	Lethality=−24.6224−0.1580·Log C−0.1225 ·P+0.7365·Tv	0.992	0.225
	$Lethality=-0.0005-0.1580\cdot Log\;C-0.0339\cdot P-0.0155\cdot Tv\;-0.0061\;\;\cdot P^{2}-0.0454\;\cdot \;{Tv\;}^{2}+0.0343\;\cdot \;P\cdot T$	0.992	0.225
	$Lethality=e^{\left(-17.5203-0.4213\;x\;Log\;C-0.1250\;\cdot \;P+0.6277\;\cdot \;Tv\right)}$	0.856	3.067
*L. innocua*	Lethality=−47.9947+0.5565·Log C−0.32·P+1.5398·Tv	0.999	0.099
	$Lethality=-0.0011-0.5565\cdot Log\;C-0.0745\cdot P-0.0340\cdot Tv\;-0.0134\;\;\cdot \;P^{2}-0.01002\;\cdot \;{Tv\;}^{2}+0.0745\;\cdot \;P\cdot \;T$	0.999	0.099
	$Lethality=e^{\left(-27.9629+0.1103\;\cdot \;Log\;C-0.1833\;\cdot \;P+0.8820\;\cdot \;Tv\right)}$	0.946	3.472
*E. coli*	Lethality=−50.1299+2.1757 · Log C−0.269 · P+1.2969 · Tv	0.998	0.102
	$Lethality=-0.0014+2.1757\cdot Log\;C-0.0969\cdot P-0.0442\cdot Tv-0.0167\cdot P^{2}\;\;-0.1316\cdot {Tv\;}^{2}+0.0949\cdot P\cdot T$	0.998	0.102
	$Lethality=e^{\left(-30.3236+2.6268\;\cdot \;Log\;C-0.0998\;\cdot \;P+0.4829\;\cdot \;Tv\right)}$	0.986	3.117
*F. helveticus*	Lethality=−40.1701+0.372·Log C−0.2779·P+1.3375·Tv	0.994	0.199
	$Lethality=-0.0008+0.372\cdot Log\;C-0.0594\cdot P-0.0271\cdot Tv-0.0107\;\cdot P^{2}\;\;-0.0796\cdot {Tv\;}^{2}+0.0597\cdot P\cdot T$	0.994	0.199
	$Lethality=e^{\left(-13.7818+0.0949\;\cdot \;Log\;C-0.0946\;\cdot \;P+0.455\;\cdot \;Tv\right)}$	0.980	3.367

Log C: quantity of inoculated microorganisms expressed as Log CFU/mL; P = pressure expressed in MPa; Tv = valve temperature expressed in °C.

**Table 4:** Immunoglobulin isotype retention in human milk after each treatment.

	Raw HM	HoP	UHPH 200	UHPH 250
sIgA (%)	100.0 ± 3.0 ^A^	50.9 ± 2.6 ^B^	83.0 ± 3.6 ^A^	90.5 ± 7.4 ^A^
IgG (%)	100.0 ± 11.7 ^A^	54.9 ± 6.9 ^B^	107.9 ± 10.2 ^A^	98.9 ± 16.0 ^A^
IgM (%)	100.0 ± 0.62 ^A^	46.5 ± 3.2 ^B^	112.8 ± 5.5 ^A^	108.7 ± 5.1 ^A^

Data of Ig isotype composition in each condition are expressed as relative proportion vs. raw HM values (%). Results are presented as the mean ± standard error of the mean. Different capital letters indicate significant differences (*p* < 0.05) between treatments. HM = human milk; HoP = Holder pasteurization; IgG = immunoglobulin G; IgM = immunoglobulin M; sIgA = secretory immunoglobulin A; UHPH = ultra-high-pressure homogenization.

## Data Availability

The datasets generated and/or analyzed during the current study are available from the corresponding authors on reasonable request. The data are not publicly available due to ongoing further research.
